# Peripheral and central nervous system involvement in a patient with primary Sjögren’s syndrome: a case report

**DOI:** 10.1186/s13256-019-2086-8

**Published:** 2019-05-25

**Authors:** Kathi Ging, Marie-Luise Mono, Mathias Sturzenegger, Martin Zbinden, Sabine Adler, Vera Genitsch, Franca Wagner

**Affiliations:** 1 Institute of Anatomy, University of Zurich, Zurich, Switzerland; 2 Department of Neurology, Stadtspital Triemli, Zurich, Switzerland; 3 Department of Neurology, Inselspital, Bern University Hospital, Bern, Switzerland; 4 Department of Rheumatology, Inselspital, Bern University Hospital, Bern, Switzerland; 5 Department of Pathology, Inselspital, Bern University Hospital, Bern, Switzerland; 6 University Institute of Diagnostic and Interventional Neuroradiology, Inselspital, Bern University Hospital, Freiburgstrasse 4, 3010 Bern, Switzerland

**Keywords:** Sjögren’s syndrome, Central nervous system, Polyneuropathy, Magnetic resonance imaging

## Abstract

**Background:**

Primary Sjögren’s syndrome is the second most common rheumatological disorder after rheumatoid arthritis. It typically presents as xerophthalmia and xerostomia in postmenopausal women. Involvement of the central nervous system has been recognized, although its pathogenesis and characteristics are poorly understood. Central nervous system complications are a diagnostic challenge and emphasize the need for systematic screening of patients with new peripheral and central neurological symptoms.

**Case report:**

We report a case of a 58-year-old Swiss woman presenting with rapidly progressive sensorimotor distal polyneuropathy together with new-onset generalized seizures. Initial magnetic resonance imaging (MRI) of the brain performed after the first seizure showed multiple, bihemispheric, confluent white matter hyperintensities with contrast enhancement. Follow-up imaging 3 days after the initial magnetic resonance imaging demonstrated a fulminant disease progression associated with the serious clinical deterioration of the patient. In light of the results of a minor salivary gland biopsy, autoantibody testing, nerve conduction studies, and cranial magnetic resonance imaging, primary Sjögren’s syndrome with cryoglobulinemia type II was diagnosed. Response to plasmapheresis and subsequent administration of cyclophosphamide was favorable.

**Conclusion:**

Even though exocrinopathy is the hallmark of Sjögren’s syndrome, systemic symptoms are observed in one-third of patients. There is an urgent need to better characterize the mechanisms underlying different disease phenotypes and to perform randomized controlled trials in order to provide tailored and evidence-based treatment for primary Sjögren’s syndrome.

## Introduction

Primary Sjögren’s syndrome (pSS) is the second most common rheumatological disorder after rheumatoid arthritis, affecting around 0.5% of the general population [1]. Females are much more often affected by Sjögren’s syndrome (SS) than males (9:1 ratio), and onset usually occurs in the fourth or fifth decade of life [2]. SS typically presents as xerophthalmia and xerostomia in postmenopausal women. Extraglandular manifestations occur in over 30% of cases, mainly affecting the joints, skin, lungs, and peripheral nervous system [3, 4].

The American College of Rheumatology (ACR) endorsed the 2012 classification criteria for pSS [5]. These criteria consist of three items: ocular staining score (OSS) ≥ 3, positive autoantibodies, and minor salivary gland biopsy focus score ≥ 1. Patients meeting two of these criteria are classified as having pSS. In 2016, the ACR and the European League Against Rheumatism (EULAR) proposed new classification criteria [6]. They adopted five objective items with different weights, giving a higher weight of 3 points each to positive autoantibody and positive biopsy results (focus score ≥ 1). OSS ≥ 5 (or van Bijsterveld score ≥ 4), Schirmer test ≤ 5 mm/5 min, and unstimulated salivary flow rate ≤ 0.1 ml/min each score 1 point. A patient with a total score ≥ 4 is deemed to have pSS [6]. Involvement of the central nervous system (CNS) has been recognized, although its pathogenesis and characteristics are varied and poorly understood [4, 7].

The first published description of CNS involvement with focal or diffuse symptoms was the series of eight patients described in 1982 by Alexander *et al.*, who suggested a direct etiopathogenetic role of the anti-Ro (anti-SSA) antibodies [8]. Because severe neuropsychiatric syndromes may occur even with the seronegative forms of pSS, we emphasize the importance for clinicians of being familiar with the extraglandular manifestations of this syndrome [9]. Furthermore, CNS involvement may precede clinical diagnosis by many years and may lead to an underestimation of other neurological and/or systemic diseases [10]. The aim of this case report is to describe the CNS signs and symptoms in a patient with severe symmetric axonal sensorimotor polyneuropathy and rapidly evolving disseminated cerebral lesions in the presence of type II cryoglobulins as the predominant finding of pSS.

## Case presentation

A 58-year-old Swiss woman presented to our hospital with a history of ascending numbness in both legs evolving over the preceding 12 months. Three weeks before initial evaluation, she had developed rapid, painless worsening of her symptoms. Her main complaints were motor weakness and loss of fine motor skills. Sicca syndrome and Raynaud’s phenomenon had been present for more than 10 years. The patient denied having joint pain. Clinical examination revealed distally accentuated, symmetric, flaccid tetraparesis with areflexia accompanied by hypesthesia up to the knees and elbows for all sensory qualities. After administration of intravenous methylprednisolone for suspected inflammatory polyneuropathy, the patient had her first generalized tonic-clonic seizure. Despite the administration of levetiracetam, another two generalized seizures occurred 24 h later, after which the patient showed psychomotor slowing, right-sided hemianopia, and central paresis of the right arm.

The initial electroencephalogram (EEG) after the first seizure showed slowing of both occipital lobes with temporal acceleration. This finding was more pronounced on the left side and over the right hemisphere. Electrophysiological studies revealed a severe axonal sensorimotor proximal symmetric polyneuropathy with sensory proximal symmetric accelerated defiance.

**Fig. 1:**
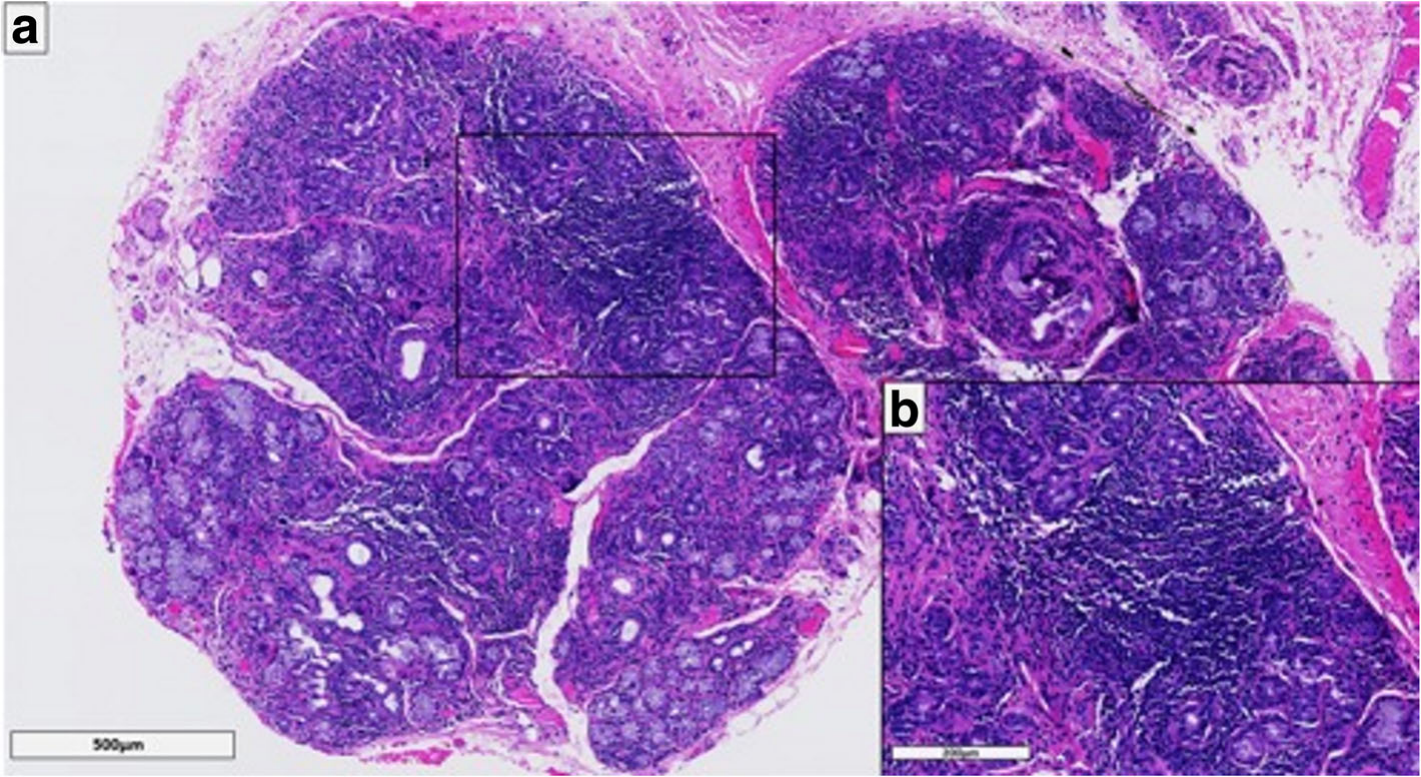
**a** and **b** Histology of minor salivary glands. **a** The histology of minor salivary glands of the lips (H&E staining) revealed diffuse, predominantly lymphocytic inflammatory infiltrates with formation of lymphocytic foci. **b** Magnification of one lymphocytic focus

Laboratory testing demonstrated rheumatoid factor, an antinuclear antibody titer of > 1:1280, and antibodies to SSA/Ro and SSB/La together with hypocomplementemia. Type II cryoglobulins were detectable (cryocrit of 5.4%). Antibodies against double-stranded DNA (deoxyribonucleic acid) were absent. Saxon and Schirmer tests confirmed severely decreased tear and saliva production. Biopsy (Fig. 1) of labial minor salivary glands showed periductal lymphocytic infiltration with a focus score > 1. On the basis of the positive anti-SSA as well as the salivary gland biopsy having a focus score > 1 and a Schirmer test < 5 mm in 5 min, the 2017 ACR-EULAR classification criteria for pSS were formally fulfilled. Because there was prominent hypergammaglobulinemia with markedly elevated light chains, a bone marrow biopsy was performed, which showed < 10% plasma cells. Flow cytometry demonstrated expansion of clonal plasma cells with restricted kappa light chains.

Primary lumbar puncture showed hypergammaglobulinemia with markedly increased light chains. Repeat lumbar punctures during the disease course confirmed monoclonal gammopathy of undetermined significance (MGUS) of the immunoglobulin M kappa type.

**Fig. 2:**
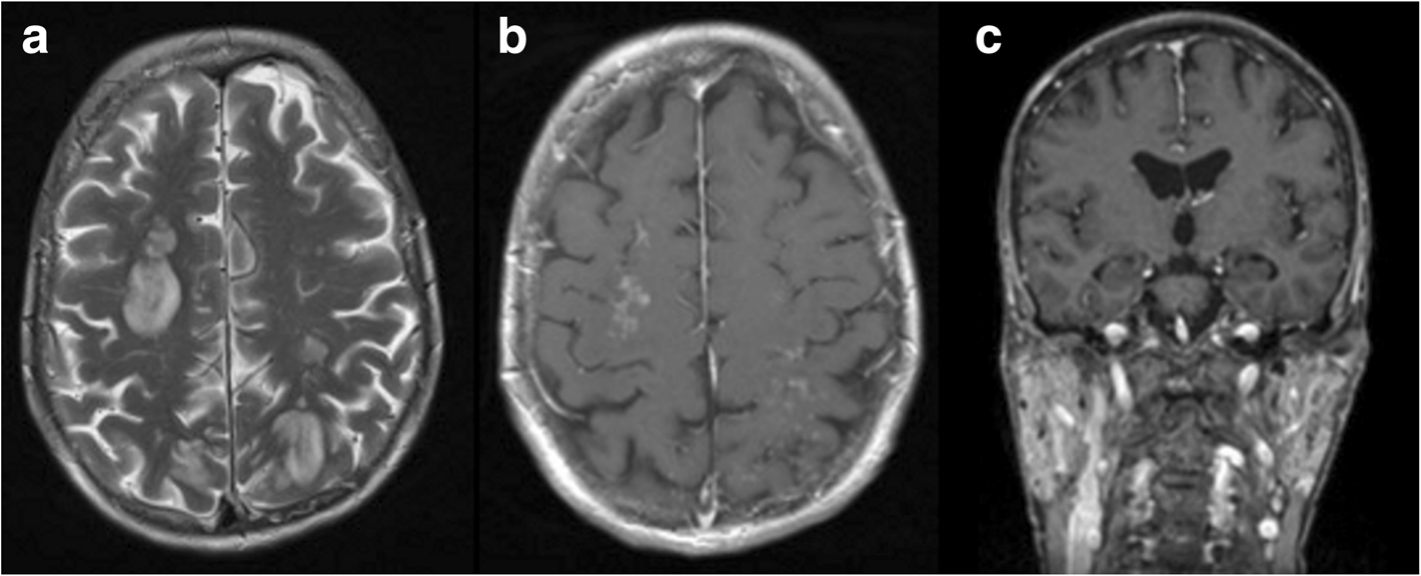
**a**–**c** Initial magnetic resonance imaging (MRI) of the brain. The initial MRI scan showed multiple disseminated, bihemispheric, supratentorial cortical, and subcortical hyperintense white matter lesions in the axial T2-weighted (3 mm) images, especially in the frontal, parieto-occipital, and occipitotemporal lobes (**a**). The white matter hyperintensities revealed contrast enhancement with a diffuse, fine granular pattern (**b**; axial T1-weighted image after contrast application, 3 mm). The parotid glands were diffusely enlarged on both sides with multiple small cystic areas and tiny contrast-enhancing nodules (**c**; coronal T1-weighted MPR after contrast application, 1 mm)

**Fig. 3:**
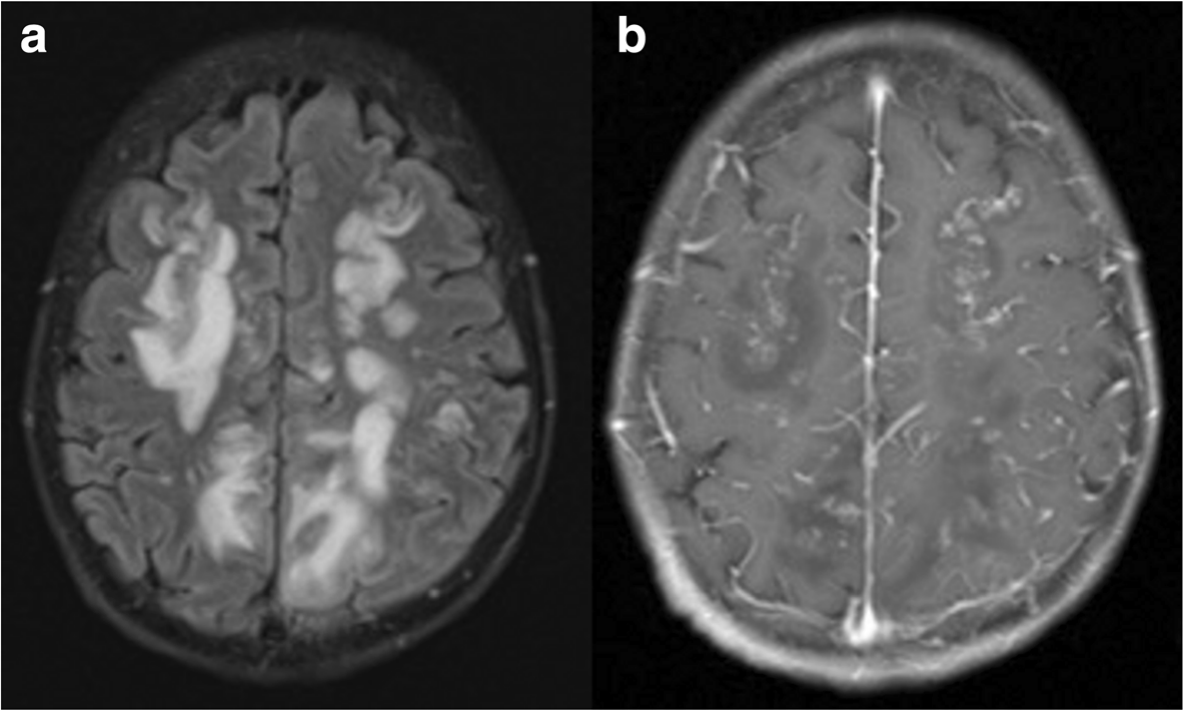
**a** and **b** Follow-up magnetic resonance imaging (MRI) of the brain. Follow-up MRI of the brain 3 days after the first scan demonstrated a significant increase in size and extension of the now-confluent white matter lesions on the axial 3-mm fluid-attenuated inversion recovery image (**a**), especially notable bilaterally in the frontal and parietal lobes with stipple contrast enhancement with a “miliar” distribution pattern (**b**; axial T1-weighted image after contrast application, 3 mm)

Initial magnetic resonance imaging (MRI) of the brain performed after the first seizure showed multiple, bihemispheric, confluent white matter hyperintensities (WMHs) with contrast enhancement (Fig. 2). The parotid gland on both sides and the left submandibular gland were diffusely enlarged with multiple small cystic areas and tiny contrast-enhancing nodules (Fig. 2). MRI was performed 3 days after the first imaging because of rapid clinical worsening, which demonstrated a fulminant disease progression (Fig. 3). Consequently, a biopsy of one of the enhancing lesions in the right frontal lobe was done. The right frontal dura and slightly thickened right pia mater were also biopsied and sent for pathological and microbiological testing. The histopathological results were noncontributory and did not suggest a specific pattern or definitive diagnosis. The possibility of microglial activation was discussed. There was no evidence of CNS vasculitis; infection with cytomegalovirus, herpes simplex virus, JC virus (human polyomavirus 2, formerly John Cunningham virus), or *Toxoplasma gondii*; or lymphoma infiltrates.

pSS was suspected in light of the patient’s sicca syndrome and results of laboratory testing. The labial minor salivary gland biopsy showed no evidence of another underlying rheumatological disorder such as systemic lupus erythematosus. The fulminant worsening of symptoms led us to consider an additional lymphoma in the course of long-standing SS as a differential diagnosis. However, progression of the CNS lesions under administration of steroids as well as the results of the bone marrow biopsy argued against this diagnosis. No evidence for an immunoglobulin G4 (IgG4)-related disease was found in the biopsy of the salivary glands, and results of serological testing for hepatitis C were negative. Results of the bone marrow biopsy and flow cytometry were interpreted as MGUS.

**Fig. 4:**
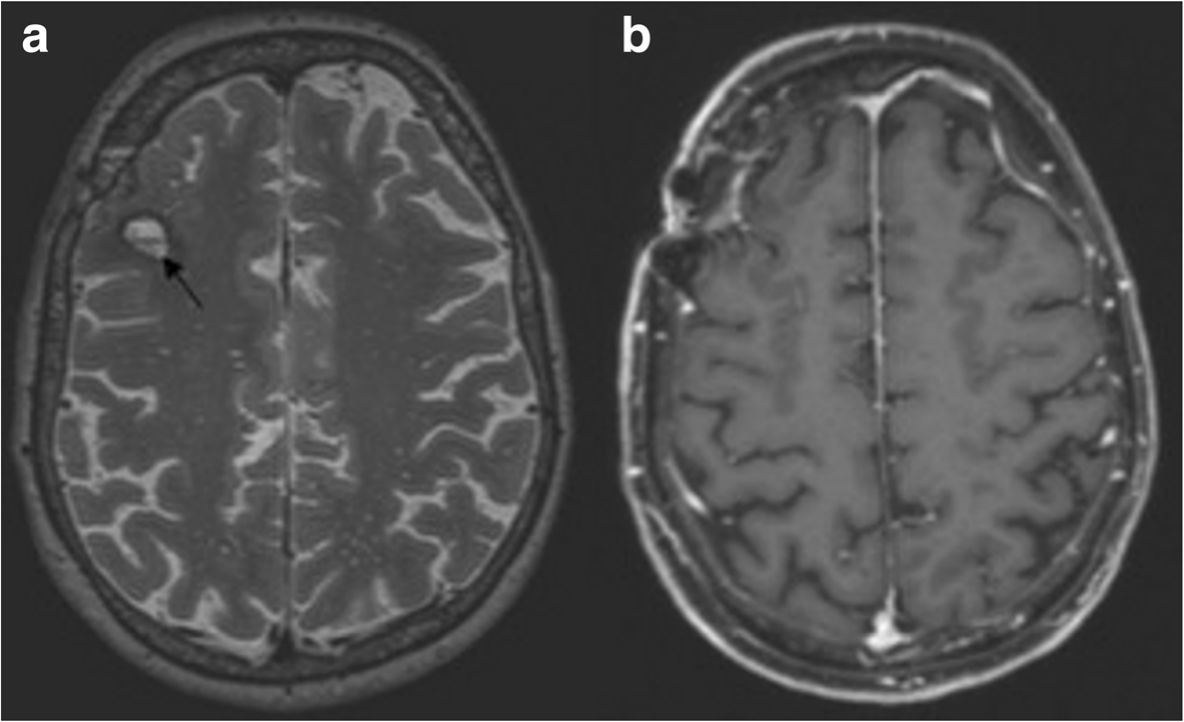
**a** and **b** Final magnetic resonance imaging (MRI) of the brain. Follow-up MRI performed 5 months after symptom onset demonstrated complete regression of the previously documented confluent white matter lesions on the axial T2-weighted (3 mm) image (**a**) and the contrast enhancement in the T1-weighted (3 mm) image after contrast application (**b**). At the site of the earlier biopsy, a small liquor isointense parenchymal defect with a T2-weighted hypointense hemosiderin rim and residual contrast enhancement of the biopsy cavity was seen in the right frontal lobe (**a**; *arrow*)

After the patient had her third seizure, five sessions of plasma exchange were conducted over 8 days. After the second generalized seizure and until plasmapheresis, the patient showed reduced vigilance and psychomotor slowing. Thereafter, cyclophosphamide was administered monthly, along with oral steroids. Plasmapheresis led to a rapid improvement of the patient’s condition. After the seventh cycle of cyclophosphamide therapy, the patient was able to walk unaided for up to 1 h and carry out everyday activities independently. Steroids were gradually tapered. MRI performed after the second cycle of cyclophosphamide demonstrated complete resolution of the contrast-enhancing WMHs (Fig. 4). Six months after initial presentation, cryoglobulins were no longer detectable. Treatment was changed to rituximab given every 6 months. Furthermore, intermittent depressive mood led to a switch of the antiepileptic therapy from levetiracetam to lamotrigine after 6 weeks. The patient is still taking lamotrigine and is seizure-free. Under therapy with lamotrigine, the patient had normal EEGs with normal basic activity and no typical epilepsy signals.

The patient recovered quickly and was able to resume everyday life within 3 months after leaving the hospital. The patient is currently doing well with no signs of relapse 1.5 years after initial presentation and has returned to work.

## Discussion

In 1933, the Swedish ophthalmologist Henrik Sjögren first described the clinical and histological features of “keratoconjunctivitis sicca” [3]. pSS is distinguished from a secondary form that occurs in the presence of another underlying systemic autoimmune disorder, such as systemic lupus erythematosus. The classical patient is a postmenopausal woman presenting with ocular and oral dryness. Fatigue and joint pain are also common, with involvement of the skin, the lungs, and the nervous system being less frequent [3]. The frequency of the neurological manifestations of pSS is difficult to assess because of the lack of studies in a broad population of patients, but it is estimated to be 20% [4, 7]. Peripheral nervous system impairment is the most common neurological finding. CNS involvement seems less frequent, and the reported manifestations are varied (*see* Table 1). A retrospective study of 82 patients with pSS on a neurology and internal medicine ward found that neurological complications were the first symptoms in 81% of patients [7]. The leading presentation is sensory neuropathy, with painful small-fiber neuropathy and sensory ataxic neuropathy being the most common phenotypes [4, 7, 11–13]. The absence of uniform definitions and the changing classification criteria as well as referral bias might account for the wide range of the estimates of prevalence and the heterogeneous manifestations of CNS involvement [7, 11]. Focal or multiple cerebral T2 hyperintensities, transverse myelitis, and optic neuritis mimicking multiple sclerosis have been described [4, 7, 11], but they are not specific for pSS. Furthermore, an association with neuromyelitis optica spectrum disorders has been observed [4].

**Table 1:** Neurological complications of primary Sjögren’s syndrome

Peripheral nervous system involvement	Central nervous system involvement
Sensory neuronopathy	Focal manifestations (for example, motor/sensory deficit)
Sensorimotor polyneuropathy	Aseptic meningoencephalitis
Mononeuritis multiplex	Myelopathy
Demyelinating polyradiculoneuropathy	Headache
Cranial nerve involvement	Cognitive disorders
Autonomic neuropathy	Mood disorders
Myasthenia gravis	Seizure
	Pyramidal signs
	Brainstem signs
	Cerebellar syndrome
	Encephalopathy
	Spinal cord involvement
	Multiple sclerosis–like disease

Different pathogenetic mechanisms could explain the complexity of nervous system involvement and the diverse clinical presentations. Vasculitis of the vasa nervorum is considered to play a key role in mononeuritis multiplex, whereas T-cell infiltration in the dorsal root ganglia has been described in sensory ataxic neuropathy. Autoantibodies and immune-complex deposition have also been proposed as an important disease mechanism [4, 7, 11–14].

As with our patient, clinical characterization is mostly based on existing neurological syndromes. Imaging characterization [15–17] or histological characterization is still insufficient to explain the pathophysiology of pSS.

For all patients with pSS who have clinical signs of CNS involvement, and in those with autoimmune diseases more generally, neuroimaging for diagnosis and follow-up should follow a standardized protocol. The role of brain MRI in the assessment of CNS involvement in pSS has not yet been fully defined. Diffuse, small, and punctate WMHs represent a frequent but nonspecific finding, and their prevalence increases with age, diabetes, atherosclerosis, and hypertension [18]. Multiple WMHs have been detected in up to 80% of patients with pSS and focal progressive neurological dysfunctions and in 50% of patients with a diffuse pattern [10, 19]. These abnormalities have been reported more frequently in asymptomatic patients with pSS than in age- and sex-matched healthy subjects [19–21]. In recent years, specific metabolic tissue markers found by magnetic resonance spectroscopy (MRS) in contrast to the presence of structural damage were described [22]. MRS provides functional information about the brain region in which metabolic ratios are measured and has been used frequently in other neuroimmunological conditions, such as neuropsychiatric systemic lupus erythematosus [23]. The somewhat nonspecific and diffuse neurological symptoms are likely to be related to an early subtle and functional impairment of the CNS due to supposed endotheliitis. Effectively, *N*-acetylaspartate (NAA) levels and the NAA/Cr ratio decrease in subcortical frontal and basal ganglia white matter detected with MRS and are known to be related to impairment of microvasculature [22]. In our opinion, MRS might detect a potential early biomarker of CNS involvement in pSS, is less expensive and less invasive than brain biopsy, and could avoid performing a brain biopsy that might be inconclusive, as in our patient. Further studies in a larger cohort of patients with pSS and CNS involvement are needed to identify patterns of brain involvement in pSS more clearly. MRI with MRS might represent an effective method for the early diagnosis of CNS involvement, particularly when neurological symptoms precede systemic involvement. Persistent disability appears to be more frequent in patients with CNS involvement than in those with peripheral nervous system involvement, suggesting the need for intensive and early treatment in cases of CNS involvement [18]. Clinicians should always consider pSS in patients presenting with polyneuropathy and/or neurological symptoms that are not typical of other autoimmune CNS disorders.

Feared complications of long-standing SS are cryoglobulinemic vasculitis and B-cell lymphoma. Delalande *et al.* detected mixed cryoglobulins in 36.6% of their patients with nervous system involvement [7]. Cryoglobulinemia is most often seen in patients with sensorimotor polyneuropathy and is associated with severe extraglandular manifestations and an increased risk of lymphoma [12].

Diagnosis requires serological (presence of SSA/Ro or SSB/La) or histological (minor salivary gland biopsy) evidence of autoimmunity. Objective testing of ocular and oral dryness should be performed (such as with Schirmer and Saxon tests). Hepatitis C virus and human immunodeficiency virus infection, lymphoma, sarcoidosis, and IgG4-related disease should be excluded [3].

Depending on disease manifestation, treatment ranges from topical agents to relieve ocular and oral dryness to administration of systemic immunosuppressive drugs in patients with organ involvement. Studies are hampered by differing classification systems and a lack of good clinical endpoints. Steroids represent the mainstay of therapy. Intravenous immunoglobulin has been reported to positively affect disease course in small-fiber and sensory ataxic neuropathy [4, 7, 11–13]. Due to their pathogenetic role, monoclonal antibodies targeting B-cells (including rituximab and belimumab) are increasingly used [24]. In life-threatening disease related to cryoglobulinemic vasculitis, plasma exchange followed by cyclophosphamide or rituximab should be considered [24]. With a deeper understanding of disease mechanisms and the establishment of prognostic markers, more tailored treatment regimens are expected to evolve.

## Conclusion

Extraglandular manifestations of pSS comprising arthralgia, skin and lung, and peripheral nervous system involvement can be observed in up to one-third of patients. Involvement of the CNS has also been recognized, although its pathogenesis and characteristics are varied and poorly understood. This case report underlines the diversity of neurological complications of pSS. The low frequency of neurological symptoms as the first manifestation of pSS, especially in the event of CNS involvement, could explain why SS is often underdiagnosed or diagnosis is delayed. Magnetic resonance screening for pSS should be systematically performed in cases of acute or chronic myelopathy, axonal sensorimotor neuropathy, or clinical suspicion of CNS involvement.

Cryoglobulinemic vasculitis should be considered if a rheumatoid factor together with hypocomplementemia can be detected in a patient with SS. Affected patients are at increased risk of developing lymphoma as well as severe systemic complications and should be closely monitored. Treatment of SS is based on the clinical symptoms and their severity. Given the rarity of CNS involvement, therapy mostly relies on expert opinion.

### Take-home points

Extraglandular manifestations of pSS as well as peripheral nervous system involvement are seen in up to one-third of patients. Involvement of the CNS has also been recognized, although its pathogenesis and characteristics are varied and poorly understood.

Cryoglobulinemic vasculitis should be considered if a rheumatoid factor together with hypocomplementemia can be detected in a patient with SS. Affected patients are at increased risk of developing lymphoma as well as severe systemic complications and should be closely monitored.

Neurological onset often precedes by many years both the appearance of systemic symptoms and the immunological diagnosis. Thus, pSS should always be considered in patients with relatively nonspecific neurological symptoms associated with sicca syndrome. MRI and neuropsychological assessment are necessary for an early diagnosis, particularly when neurological symptoms precede systemic involvement. Because of the great heterogeneity of neurological involvement in pSS, neurological complications should be the subject of large prospective cohort studies.

Treatment of SS is based on clinical symptoms and their severity. Given the rarity of CNS involvement, therapy mostly relies on expert opinion.

## Abbreviations

ACR: American College of Rheumatology
CNS: Central nervous system
Cr: Creatinine
EEG: Electroencephalogram
EULAR: European League Against Rheumatism
IgG4: Immunoglobulin G4
MGUS: Monoclonal gammopathy of undetermined significance
MRI: Magnetic resonance imaging
MRS: Magnetic resonance spectroscopy
NAA: *N*-acetylaspartate
OSS: Ocular staining score
pSS: Primary Sjögren’s syndrome
WMH: White matter hyperintensity
